# The Mouth and the Teeth. American Health Primer

**Published:** 1880-01

**Authors:** 


*The Mouth and the Teeth.* By J. W. White, M. D., D. D. S. This is another of the series pertaining to Sanitary Science and the preservation of the health, known as American Health Primers, edited by W. W. Keen, M. D., and published by Lindsay & Blackiston, Philadelphia.

This volume by Dr. White is an interesting and comprehensive treatise, giving valuable information and directions for the preservation of the teeth and adjacent parts. It is illustrated by engravings fully explaining the text, and must prove of essential service to all interested in the care of children, as well as in the condition of their own dentures. Commencing with the Mouth we find the teeth from their development to their eruption fully described, together with their nervous relations, constitutional peculiarities and defects. The hygiene of the mouth receives careful notice, and artificial dentures closes the subjects treated of. So much that is useful is embraced in this small work, and so clearly and correctly is all explained, that it forms one of the most important volumes of the series. The price of these volumes is but 50 cents.

